# Intolerance of uncertainty and negative emotions among high school art students during COVID-19 pandemic: a moderated mediation analysis

**DOI:** 10.3389/fpubh.2024.1277146

**Published:** 2024-05-22

**Authors:** Congcong Fu, Jin Liu, Baojuan Ye, Qiang Yang

**Affiliations:** ^1^Center of Mental Health Education and Research, School of Psychology, Jiangxi Normal University, Nanchang, Jiangxi, China; ^2^School of Music, Jiangxi Normal University, Nanchang, Jiangxi, China; ^3^Center of Mental Health Education and Research, School of Education, Jiangxi Normal University, Nanchang, Jiangxi, China

**Keywords:** intolerance of uncertainty, psychological capital, family functioning, negative emotions, COVID-19, high school art students

## Abstract

**Objectives:**

A number of high school art students experience negative emotions during their preparation for the art college entrance examination, characterized by worries and fear of uncertainty. Therefore, how individual difference factors, such as intolerance of uncertainty, affect the negative emotions of students needs to be examined. Inspired by the integrative model of uncertainty tolerance, the current study seeks to explain the association between intolerance of uncertainty and negative emotions by testing the potential mediating role of psychological capital and the moderating role of family functioning.

**Patients and methods:**

A total of 919 Chinese high school art students (*M*_age_ = 18.50 years, range = 16–22) participated from November 2022 to December 2022. Convenience sampling strategies were used. The participants were asked to complete the measures of intolerance of uncertainty scale, psychological capital questionnaire, depression anxiety stress scale, and family adaptability and cohesion evaluation scale. The data were analyzed using Pearson’s r correlations and moderated mediation analysis.

**Results:**

Results showed that intolerance of uncertainty was positively associated with negative emotions but negatively associated with psychological capital, which in turn, was negatively associated with negative emotions. Psychological capital mediated the indirect link of intolerance of uncertainty with negative emotions. Family functioning buffered the impact of psychological capital on negative emotions.

**Conclusion:**

This study can enhance our understanding of the intolerance of uncertainty on negative emotions and provide insights on interventions for high school art students’ negative emotions for educators. The interventions targeting intolerance of uncertainty, psychological capital and family functioning may be beneficial in reducing the effect of intolerance of uncertainty on negative emotions faced by high school art students.

## 1 Introduction

Education has been highly valued in China since classical times (1). China established a feudal imperial exam system to select government officials in the early Sui Dynasty (606 AD) (2). In China, The National College Entrance Examination, a difficult exam, is administered every June (3). Students all over the country take the exam organized by the government on 3 days. The examination consists of three compulsory subjects (mathematics, Chinese, and English) and optional subjects (politics, physics, biology, chemistry, history, and geography) (1). The group of students taking the examination can be divided into four categories: Liberal arts students, science students, art students and physical education students (4). For high school art students, they not only take the examination of compulsory subjects and optional subjects administered every June, but also take the art examination administered every December before the examination of compulsory and optional subjects. The overall test score is the determinant of whether and which college the student will attend (1). There are three cut-off scores dictated by the Ministry of Education. One decides entrance to prestigious universities. The second is the minimum score for entry into ordinary universities, and the third is the score for entry into higher vocational colleges. Moreover, some universities, especially art colleges, are qualified to organize examinations separately. Students need to apply to the college first, and then go to the college to take the exam.

COVID-19 is a global health threat (5). Studies have shown that COVID-19 has had a negative effect on the mental health of different groups of people (6–9). A large number of individuals have been forced to rapidly transition to a new way of life because of COVID-19 (10). In China, the COVID-19 pandemic gave rise to major challenges in the implementation of education programs. Many high schools were required to move completely to online teaching methods. In the wake of the COVID-19 pandemic, some regions have taken several measures to guarantee the health of students, such as school suspensions and keeping social distance guidelines. However, some art students needed to take an exam at the university they were applying to. In China, the Ministry of Education issued a notice that strict and mandatory measures must be taken by colleges to control the spread of the epidemic. Some colleges, such as Communication University of China and Beijing Film Academy, announced the use of online examinations to ensure the health of high school art students. Therefore, some students had to prepare actively for this new form of examination, and this change in the examination method gave rise to feelings of uncertainty related to examination performance among the students. In addition, during the COVID-19 pandemic, the teachers provided feedback on the students’ homework through online channels. These changes led to additional workload and stress on high school art students. The learning and examination processes of high school art students became riddled with uncertainty. The impact of these uncertainties on the physical and mental health of students has aroused wide concern in society.

Probability is a major source of uncertainty (11). Both the pandemic and students’ performance in the examination are subject to probabilities. Numerous studies in multiple disciplines have reported the effects of uncertainty on anxiety, worry, and avoidance of decision-making (12). According to previous research, individuals apply various responses to uncertainty, and understanding these individual differences is a significant focus of future studies (12). Thus far, however, few studies have investigated the roles of psychological capital (PsyCap) and family functioning in the relationship between intolerance of uncertainty (IU) and negative emotions among Chinese high school art students. The current research aimed to investigate the association between IU and negative emotions among high school art students, the mediating role of PsyCap, and the moderating role of family functioning in this correlation.

### 1.1 Intolerance of uncertainty and negative emotions

Intolerance of uncertainty (IU) refers to a cognitive bias, and this cognitive bias can influence how an individual perceives, interprets, and responds to uncertain situations at cognitive, emotional, and behavioral levels (13, 14). Kurita et al. (15) studied the effects of IU on patients with lung cancer. They found that IU was significantly associated with high levels of non-somatic depressive symptoms and perceived stress (15). Likewise, Demirtas and Yildiz (16) found that IU was significantly associated with perceived stress among college students in Turkey. Similarly, Huntley et al. (17) examined the students’ perceived level of IU self-developed questionnaire, and the results suggested a positive association between IU and test anxiety among college students in the UK. In addition, the integrative model of uncertainty tolerance hypothesizes that individuals develop a set of cognitive, emotional, and behavioral responses to uncertainty of external stimuli, such as threat, worry, and despair (12). In our study, the depression anxiety stress scale was applied to measure negative emotions in three dimensions: depression, anxiety, and stress (18).

The COVID-19 pandemic has led to uncertainties in the learning and examination processes of high school art students. The symptoms of SARS-CoV-2 onset can also influence the performance of high school art students at their examination. For most high school art students, art college entrance examination is a life-changing event, and it is difficult to foresee the examination activities. Indeed, high school art students with IU are likely to interpret uncertain information as threatening (19), contributing to significant somatic stress reactions (20, 21). Previous studies have shown that a key cognitive distinction associated with anxiety is a “sense of uncontrollability focused on the possibility of future threat, danger or other potentially negative events” (22). In addition, IU has been hypothesized to prompt individuals to engage in over-identification of potential problems and negative orientations (14). In other words, high school art students with IU strongly believe that they will fail the exam. When high school art students are asked to mention the reasons that underlie their fear of failure, the students with IU provide more reasons than those without IU. As a special group in high school, high school art students are expected to engage in both cultural learning and professional training simultaneously. Therefore, individuals with high level of IU experience high levels of depression, anxiety, and stress during the pandemic. However, the impact of the relationship between IU and negative emotions of students during their preparation for an art college entrance examination is still unknown. The COVID-19 offers us an opportunity to investigate the relation between IU and negative emotions under a naturally stressful environment.

The theoretical and practical implications of the study are as follows: First, the majority of recently published studies on IU have focused on the effect of IU on emotion, behavior, and belief. For example, they have examined the effect of IU on social anxiety (23), compulsive exercise (24), and loss-related risk preference (25). However, to our knowledge, few studies have explored what mediates the association between IU and negative emotions and what influences the direction or strength of this relationship. The novelty of this study lies in the fact that, to our knowledge, it is the first to explore the mediating and moderating roles of PsyCap and family functioning on the correlation between IU and negative emotions. Second, recent studies on IU have focused on a variety of groups and situations, such as the effect of IU among preservice teachers (26), autistic pupils (27), and breast cancer patients (28). To our knowledge, no study has explored the association between IU and negative emotions of high school art students preparing for entrance examination in the context of COVID-19, given its novelty. Previous studies have shown that art students reported higher rates of anxiety and depression symptoms compared to the general student population (29). Zilinskas and Lesinskiene (30) examined the difference in suicidal ideation among students of different majors, and the results suggested art students expressed the highest levels of suicidal ideation. Compared to students of other majors, art students are more likely to have lower mood (31). High school art students experience a variety of negative emotions such as depression, anxiety, and stress when preparing for art college entrance examination. However, much less attention has been paid to the mental health status of the National College Entrance Examination population, especially for art students. Therefore, in order to fill the knowledge gap and help students reduce negative emotions, it is vital to examine the factors related to negative emotions experienced by high school art students who participated in the National College Entrance Examination in the context of COVID-19. Consequently, we propose the following hypothesis:

*Hypothesis 1*: Intolerance of uncertainty is positively related to Chinese high school art students’ negative emotions.

### 1.2 Psychological capital as a mediator

Psychological capital (PsyCap) is conceptualized as an individual’s positive psychological state of development, and it comprises four components: hope, optimism, resilience, and self-efficacy (32). Turliuc and Candel (33) examined the relationship between PsyCap and some indicators of mental health (depression, anxiety, satisfaction with life) in a longitudinal study of 290 participants. The found that PsyCap impacts depression, anxiety, and stress in a significant way (33). A previous study also showed that IU was negatively correlated with PsyCap (34). The integrative model of uncertainty tolerance hypothesizes that individuals’ responses to uncertainty of external stimuli are varied, including both positive and negative responses, such as hope or despair, faith or doubt, confidence or vulnerability, and courage or fear (12). According to previous studies, high school art students with high IU tend to be pessimistic about the future (35, 36). IU can impair high school art students’ problem-solving skills (37), and prompt them to believe they cannot cope with the difficulty (38). In other words, IU can influence the self-efficacy of high school art students, and students with high IU do not have the ability to adapt to changes in learning and examination methods, resulting in the activation of negative emotions.

The conservation of resources theory hypothesizes that PsyCap is a kind of vital personal resource that can help individuals cope with emotional problems and reduce their negative consequences (39). Psychological strengths are important resources in coping with adverse and uncertain situations (40, 41). Furthermore, individuals have different psychological resources, resulting in variations in their psychological wellbeing (42, 43). Epitropaki (44) examined the role of positive PsyCap as a critical mechanism for managing employment uncertainty and found that PsyCap mediated the association between employment uncertainty and stress (44). Therefore, it is rational to deduce that PsyCap mediates the relationship between IU and negative emotions.

The location of the COVID-19 outbreak is highly unpredictable, and an examination is full of uncertainties. This study discusses the impact of IU on negative emotions of high school art students preparing for college entrance examination in the context of the current pandemic. Moreover, considering the integrative model of uncertainty tolerance and conservation of resources theory as the theoretical foundations, we examined whether PsyCap serves as a mediator between IU and negative emotions. To our knowledge, no previous study has examined this association. The present research aims to fill this gap by investigating the correlations among IU, PsyCap, and negative emotions among Chinese high school art students. Therefore, the following hypothesis emerged:

*Hypothesis 2*: Psychological capital mediates the association between intolerance of uncertainty and negative emotions among Chinese high school art students.

### 1.3 Family functioning as a moderator

Low levels of PsyCap may lead to an increase in high school art students’ negative emotions; however, not all students with low PsyCap are bound to develop negative emotions (45). Therefore, it is essential to examine the factors that impact the association between PsyCap and negative emotions. Because the Chinese government mandated home quarantine during the COVID-19 pandemic, some Chinese high school students had to stay at home with their families. The ecological model hypothesizes that environment influences the development of individuals, and the family as a microsystem plays a vital role in the mental health of individuals (46). This means the family-related variables may moderate the relationship between PsyCap and negative emotions.

Family functioning is defined as the ability of a family to function efficiently to meet basic needs and manage conflicts (47). The family system theory hypothesizes that the overall function of the family system is positively associated with the psychological state of its members, which can lead to a reduction in emotional problems (48). It indicates that there is a negative relationship between family functioning and negative emotions. In addition, family functioning is closely associated with negative emotions (49). The protective-enhancing hypothesis holds that family functioning (a protective factor) can enhance PsyCap’s (another promotive factor) effect on mental health (50). Bandura (51) affirmed that the self-efficacy beliefs of children can be developed with the help of environmental factors that enable children to adapt to their environments. In addition, Bandura also emphasized the significance of social persuasion and modeling in enhancing self-efficacy beliefs (52). Moreover, self-efficacy has been hypothesized to be an important dimension of PsyCap (53), which indicates that family functioning may have an impact on how PsyCap works. Therefore, family functioning may diminish the effect of PsyCap on negative emotions. Furthermore, PsyCap is a personal resource, and intimacy with one or more family members is a contextual resource (39). The conservation of resources theory hypothesizes that resources are linked with other resources, and the process of “gain spiral” leads to resource accumulation (54). Further, new resources can be generated by pre-existing resources (55), meaning family functioning and PsyCap can enhance each other. Therefore, it is logical to assume that the protective effect of PsyCap on negative emotions is greater for the students with high family functioning than for those with low family functioning. Moreover, previous studies have shown that family functioning significantly influences PsyCap (56, 57).

In China, high school students usually live with their parents. Previous research has shown that Chinese parents tend to control their children more than American parents (58). American parents tend to encourage independence than Chinese parents (58). Moreover, Chinese parents tend to attach importance to academic achievement more than American parents (58). Chinese parents view academic achievement as a means to acquire personal advancement, higher social status and respect in Chinese society (59). Chinese parents usually provide more care and support to students during their preparations for the National College Entrance Examination. In addition, it is a tradition for parents to accompany their children to the exams and to wait outside the exam room (1). Therefore, the level of family function may be higher during the period when students prepare for the National College Entrance Examination. The National College Entrance Examination offers us an opportunity to investigate the relation between family functioning and negative emotions.

Halbesleben et al. (60) also called for attention to the interactions between multiple resources and the effects of environmental factors on these interactions. This study extends the conservation of resources theory by considering the impact of the interaction between family functioning and PsyCap on negative emotions. Therefore, with conservation of resources theory, family system theory, and protective-enhancing hypothesis as the theoretical foundations, we examine whether the relationship between PsyCap and negative emotions is stronger for high school art students who report high family functioning. As far as we know, no prior study has investigated the moderating role of family functioning in the correlation between PsyCap and negative emotions. The present research aims to fill this gap by investigating this aspect in the context of Chinese high school art students. Therefore, the following hypothesis is proposed:

*Hypothesis 3*: Family functioning moderates the relationship between psychological capital and negative emotions among Chinese high school art students.

### 1.4 The present study

**Figure 1:**
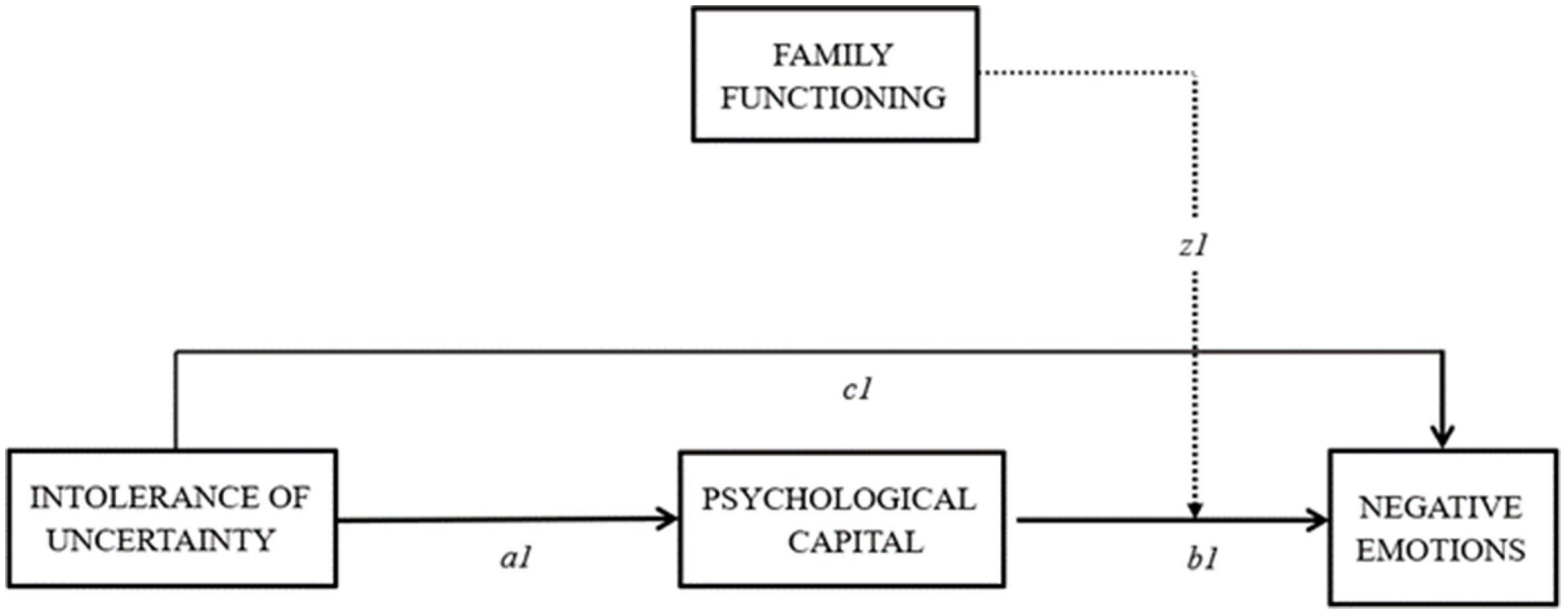
The conceptual moderated mediation model.

This study explored whether PsyCap mediates the association between IU and negative emotions (Figure 1). Moreover, this study also explored the moderating effect of family functioning on the indirect path (path z1) in this model.

## 2 Methods

### 2.1 Participants

The ethics committee of the first author’s university approved this study, and all participating high school art students provided informed consent. A total of 1,062 Chinese high school art students participated from November 2022 to December 2022 (i.e., during preparations for the art college entrance examination). Convenience sampling strategies were used. Final analyses consisted of 919 participants (*M*_age_ = 18.50, *SD*_age_ = 2.16, 46.14% female) after excluding invalid subjects (i.e., quick response or other errors).

### 2.2 Research instruments

#### 2.2.1 Intolerance of uncertainty scale

IU was measured using the intolerance of uncertainty scale (IUS-12) (14). The IUS-12 can be used as an effective tool in the research on IU in the Chinese population and has good reliability and validity (61). This scale includes 12 items (e.g., “When I am uncertain, I cannot function well”) in two dimensions: predictive anxiety and inhibitive anxiety. All items are scored on a 5-point scale ranging from 1 (*Not at all characteristic of me*) to 5 (*Entirely characteristic of me*). The alpha reliability for this sample was 0.90.

#### 2.2.2 Psychological capital questionnaire

PsyCap was assessed using the positive psychological capital questionnaire (PPQ) (62). The PPQ includes 26 items (e.g., “I feel very hopeful about the future”) in four dimensions: optimism, hope, resilience, and self-efficacy. All items are scored on a 7-point scale ranging from 1 (*Completely nonconforming*) to 7 (*Completely conforming*). The alpha reliability for this sample was 0.93.

#### 2.2.3 Family functioning scale

Family functioning was assessed using the family adaptability and cohesion evaluation scale (63). This scale can be used as an effective tool in the research on family functioning in the Chinese population and has good reliability and validity (64). The scale consists of 20 items (e.g., “Family members are very close to each other”) in two dimensions: cohesion and adaptability. All items are scored on a 5-point scale ranging from 1 (*Never*) to 5 (*Always*). The alpha reliability for this sample was 0.92.

#### 2.2.4 DASS-21 scale

Negative emotions were assessed using the depression anxiety stress scale (18). The DASS-21scale can be used as an effective tool in the research on negative emotions in the Chinese population and has good reliability and validity (65). The DASS-21 consists of 21 items (e.g., “I feel sad”) and in three dimensions: depression, anxiety and stress. All items are scored on a 4-point scale ranging from 0 (*did not apply to me at all*) to 3 (*applied to me very much*). The alpha reliability for this sample was 0.97.

### 2.3 Data analysis

Data were analyzed using SPSS25. First, descriptive statistics and correlation analysis were used. Second, PROCESS Model 4 for SPSS was employed to test the mediating role of PsyCap, and PROCESS Model 14 was applied to test the moderating role of family functioning in the relationship between PsyCap and negative emotions based on the bias-corrected percentile bootstrap method (5,000 samples) (66).

## 3 Results

### 3.1 Common method bias test

In this study, the self-rating scale was used to collect the data, and the common method bias was controlled by testing at a certain interval; confidentiality of participants’ questionnaire information was guaranteed. Harman single factor test was used to test the common method bias of all the items in four variables: IU, PsyCap, family functioning, and negative emotions. Eleven factors had eigenvalues greater than 1, and the first factor explained 26.89% of the total variation, which was less than the critical value of 40% (67). Hence, there was no significant common method bias in this study (68).

### 3.2 Preliminary analysis

The means, standard deviations, and correlations are listed in Table 1. IU was positively associated with negative emotions. IU was negatively associated with PsyCap and family functioning. Family functioning was positively associated with PsyCap. Negative emotions were negatively associated with family functioning and PsyCap.

**Table 1:** Descriptive statistics.

	*M*	*SD*	1	2	3	4
Family Functioning	3.48	0.61	1			
IU	2.66	0.85	−0.08^***^	1		
PsyCap	4.50	0.82	0.31^***^	−0.27^***^	1	
Negative Emotions	0.72	0.61	−0.25^***^	0.46^***^	−0.50^***^	1

^*^p < 0.05, ^**^p < 0.01, ^***^p < 0.001, the same below. IU, Intolerance of Uncertainty; PsyCap, Psychological Capital.

### 3.3 The mediating role of PsyCap

We analyzed the data after adding gender, only child status, and monthly household income as covariates. The result showed that females tended to show lower negative emotions (see Model 3 and Model 4 of Table 2) and PsyCap (Models 2 of Table 2) than males. Monthly household income was positively associated with PsyCap (Models 2 of Table 2). To test Hypothesis 1, linear regression analysis of SPSS was utilized. The results verified Hypothesis 1 and showed that IU was significantly related to negative emotions after controlling for gender, only child status, and monthly household income (*β* = 0.46, *p* < 0.001, see Model 1 of Table 2). Moreover, according to Hypothesis 2 of this study, PsyCap would serve as a mediator of the relationship between IU and negative emotions. Model 4 of the PROCESS macro (66) was utilized to test this hypothesis. The results suggested a negative relationship between IU and PsyCap (*β* = −0.29, *p* < 0.001; Model 2 of Table 2). IU was positively associated with negative emotions (*β* = 0.35, *p* < 0.001; Model 3 of Table 2). PsyCap was negatively associated with negative emotions (*β* = −0.41, *p* < 0.001; Model 3 of Table 2) after controlling for gender, only child status, and monthly household income. Accordingly, PsyCap mediated the effect of IU on negative emotions (*a1b1* = 0.12, 95% *CI* [0.08, 0.15], *p* < 0.001), accounting for 24.76% of the total effect. Thus, Hypothesis 2 was verified.

**Table 2:** Linear regression models.

	Model 1(NE)	Model 2 (PsyCap)	Model 3 (NE)	Model 4 (NE)
Predictors	*β* (95% CI)	*β* (95% CI)	*β* (95% CI)	*β* (95% CI)
Gender	−0.10 [−0.22, −0.02]	−0.16^*^ [−0.29, −0.04]	−0.17^**^ [−0.27, −0.06]	−0.17^**^ [−0.27, −0.07]
Only child status	0.07 [−0.01, 0.26]	−0.10 [−0.24, 0.05]	0.09 [−0.04, 0.21]	0.07 [−0.05, 0.19]
Monthly household income	0.00 [0.02, 0.02]	0.03^**^ [0.01, 0.05]	0.01 [−0.01, 0.03]	0.01 [−0.01, 0.03]
IU	0.46^***^ [0.40, 0.52]	−0.29^***^ [−0.35, −0.22]	0.35^***^ [0.29, 0.40]	0.34^***^ [0.29, 0.39]
PsyCap			−0.41^***^ [−0.46, −0.35]	−0.40^***^ [−0.45, −0.34]
FF				−0.10^***^ [−0.15, −0.05]
PsyCap × FF				0.07^**^ [0.02, 0.11]
*R^2^*	0.22	0.09	0.37	0.38
*F*	63.63^***^	23.47^***^	106.33^***^	81.16^***^

Gender: 1 = male, 2 = female; Only child status: 1 = only child, 2 = non-only child; Monthly household income: from 1 (less than RMB 2,000) to 10 (greater than RMB 10,000). FF, Family Functioning; IU, Intolerance of Uncertainty; PsyCap, Psychological Capital; NE, Negative Emotions.

### 3.4 The moderating role of family functioning

**Figure 2:**
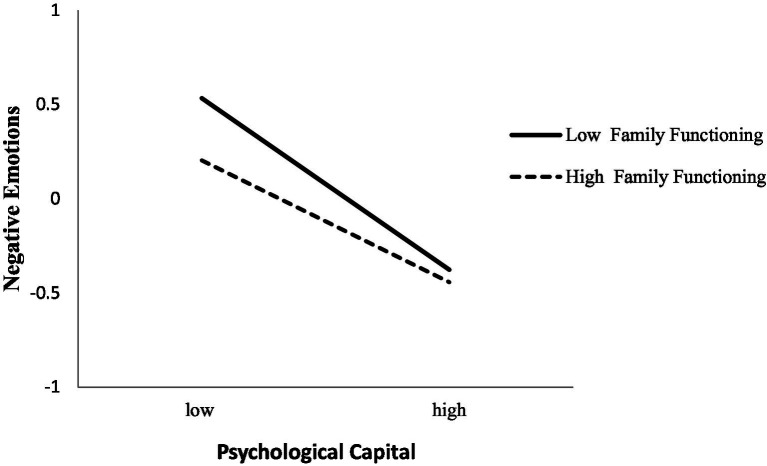
The interactive effect of psychological capital and family functioning on negative emotions.

To test Hypothesis 3, Model 14 of the PROCESS macro (66) was utilized. The results showed a significant effect of the interaction between family functioning and PsyCap on negative emotions (see Model 4 of Table 2). The specific interaction effect is shown in Figure 2. Simple slope tests showed that PsyCap had a significantly negative effect on negative emotions evoked in the presence of high and low family functioning. The effect of PsyCap on negative emotions was weaker for high school art students with high family functioning (*b_simple_* = −0.32, *t* = −10.13, *p* < 0.001) than for those with low family functioning (*b_simple_* = −0.46, *t* = −10.94, *p* < 0.001).

The bias-corrected percentile bootstrap analyses further revealed that the indirect effect of IU on negative emotions through PsyCap was moderated by family functioning. Specifically, the indirect effect of IU on negative emotions was significant in those with high family functioning (*β =* 0.09, 95% *CI* = [0.06, 0.13]) and slightly stronger in those with low family functioning (*β =* 0.13, 95% *CI =* [0.10, 0.17]). Thus, Hypothesis 3 was supported.

## 4 Discussion

To analyze the mechanisms underlying the relationship between IU and negative emotions among high school art students, the present study tested a moderated mediation model. Overall, the results suggested a positive association between IU and negative emotions. The results also showed that PsyCap partially mediated this association. Furthermore, the present study tested a moderated mediation model, and the results suggested that the association between IU and negative emotions was moderated by family functioning. This study offered empirical proof about reducing the negative emotions of high school art students during the COVID-19.

### 4.1 The relationship between IU and negative emotions

Our results verified that IU is positively related to negative emotions, consistent with the results of previous studies (6, 69). As the integrative model of uncertainty tolerance suggests, individuals with high IU could trigger a variety of appraisals and responses, such as negative emotions (12). Moreover, prior studies suggested that IU plays a transdiagnostic role across many emotional disorders (70–73). Especially during the COVID-19 epidemic, the outbreak of the pandemic and the performance of the examination led to many uncertainties, and the college application is a life-changing opportunity for many students. Individuals with IU tend to have a pessimistic outlook about future events (35, 36). In other words, high school art students with IU tend to overestimate the probability and seriousness of failure in examination, which may predispose them to threats and negative emotions.

Compared to previous studies (74, 75), our study found that IU levels were higher during students’ preparation for the art college entrance examination than other periods. In China, parents tend to hold high expectations with regards to education (1). In Chinese culture, filial piety, emphasizing the support of children to their parents, is regarded as a core value and an important social norm. In this context, the offspring generation needs to obey the authority of their parents. A previous study showed that Chinese students were more willing to accept their parents’ advice and conform to parents’ expectations of academic achievement compared to American students (76). And failure in the Examination is traditionally associated with individual and family shame (1). Moreover, in China, the National College Entrance Examination is a life-changing opportunity for many students. Failure in the examination can have very serious negative consequences. Therefore, students experienced higher levels of IU. In addition, art students tend to have higher rates of anxiety and depression symptoms compared to the general student population (29). Therefore, educators need to offer interventions that can reduce the negative emotions among art students during preparations for art college entrance examination.

### 4.2 Mediating effect of PsyCap

Further, females tended to show lower PsyCap, similar to the results of previous studies (77). Our results also showed that females tended to report lower negative emotions than males, contrary to previous research findings (78, 79). The possible reason is that male art students are more sensitive to stressful events than female students during preparations for art college entrance examination, resulting in more negative emotions. Future research is needed to examine the mediating variables in this relationship. In addition, monthly household income was positively associated with PsyCap.

Moreover, PsyCap partly mediated the effect of IU on negative emotions. Students with higher IU tended to have lower PsyCap, which may have increased the risk of negative emotions generation. With regard to the first stage of the mediation process (i.e., IU → PsyCap), this study indicated that IU was negatively associated with PsyCap. Consistent with current findings, previous studies found that IU is negatively linked with positive psychological qualities like resilience (80), optimism (81), hope (80), and PsyCap (34). During the COVID-19 epidemic, the discussion of the pandemic was shrouded in uncertainty, and the uncertainty may have prompted art school students to ruminate over possible situations.

IU refers to a cognitive bias, and this cognitive bias can influence the way that the individual responds to uncertain situations (13, 14). Individuals with generalized anxiety disorder tend to engage in chronic worrying in an effort to control feelings of uncertainty about the future (14). In addition, IU also prompts to individuals engage in over-identification of potential problems and negative problem orientation (14). As a result, this biased cognition style may reduce students’ self-efficacy and optimism. In other words, the students with high IU tend to have low PsyCap. Moreover, past research has found that PsyCap has a strong impact on negative emotions (33). In summary, during the COVID-19 outbreak and preparation for the art college entrance examination, IU was not only directly associated with negative emotions but also indirectly associated with negative emotions moderated by PsyCap.

Specifically, high school art students experienced a variety of uncertainties during the epidemic, including prolonged duration of epidemic, location of COVID-19 outbreak, symptoms of COVID-19 onset, and performance of the examination. IU may prompt students to underestimate their ability to overcome potential problems and overestimate the effect of adverse outcomes. Students with IU focus on uncertain events and psychologically associate words like “COVID-19” and “examination” with negative consequences, which in turn decreases their PsyCap. Therefore, they may experience negative emotions during their preparation for art college entrance examination in the backdrop of the COVID-19 pandemic. To our knowledge, this is the first study on the association between IU and negative emotions faced by high school art students when preparing for art college entrance examination and the mediating role of PsyCap in the association between IU and negative emotions. This mediating role has important practical significance; it suggests that the impact of IU on negative emotions is complex. PsyCap is a vital personal resource that can be reduced by IU, and this can have an impact on students’ negative emotions. The mediating effect of PsyCap provides a theoretical basis for reducing negative emotions among high school art students by improving their PsyCap.

### 4.3 The moderating effect of family functioning

Our results verified that family functioning moderates the effect of PsyCap on negative emotions faced by high school art students. As the organism-environment interaction model suggests, individuals and the environment are complex systems in which the elements do not act independently but depend on each other (82). Therefore, the influence of individual factors (e.g., PsyCap) may be changed by environmental factors (e.g., family functioning). The present study explored not only the effect of PsyCap but also of individual variations on negative emotions experienced by students during COVID-19. The results did not support the protective-enhancing hypothesis (50) suggesting that the protective impact of PsyCap on negative emotions may be greater in individuals with high family functioning than in those with low family functioning. However, the results supported the protective-antagonistic hypothesis (83) suggesting that the link between PsyCap and negative emotions was weaker in individuals with high family functioning than in those with low family functioning. There are two possible explanations for the results. First, this finding illustrates that external family-based resources may interact with internal personal resources, and individuals in low family functioning may depend more on individual self-enhancing and self-regulatory mechanisms (e.g., PsyCap). Similarly, a previous study showed that individuals with low family support had increased self-efficacy (84). Thus, individuals with high family functioning may depend less on PsyCap. Second, for individuals with high family functioning, the probability of having negative emotions is extremely low, and the protective effect of PsyCap on negative emotions may not have been apparent because negative emotions as an outcome variable showed a “floor effect.” However, for individuals with low family functioning, the “floor effect” disappeared, and the protective effect of PsyCap on negative emotions could be observed. Consistent with the current results, a previous research showed that the link between IU and negative emotions weakened as the level of hope increased (7). Other studies have shown that the effects of self-efficacy on performance may be weakened under some conditions (85, 86). In addition, previous research has also suggested that the protective effect of attachment on self-harm may not be apparent in individuals with high family self-concept (83). Given these explanations, it is not surprising that the relationship between PsyCap and negative emotions was weaker for the students with high family functioning than for those with low family functioning.

The present study examined the effect of IU and individual variations on negative emotions experienced by students during the COVID-19. To reduce negative emotions among students preparing for art college entrance examination in the backdrop of the COVID-19 pandemic, it is crucial to improve family functioning or cultivate PsyCap. Our research showed that improving PsyCap or family functioning can reduce negative emotions to a greater extent in students with low family functioning or PsyCap than in those with high family functioning or PsyCap. Therefore, we should focus more attention on interventions for students with low PsyCap or family functioning. Moreover, support from the family can help individuals cope with the threat of major events. In order to ensure the mental health of students in the face of stressful events, we need to focus on improving PsyCap and family functioning in their daily lives.

### 4.4 Implications

The theoretical significance of this paper is reflected in the following three aspects: First, the majority of recently published studies on IU have focused on the effect of IU on emotion, behavior, and belief. For example, they have explored the effect of IU on climate change distress (87), attitude toward violence against women (88), and loss-related risky preference (25). However, few studies have explored the mediating and moderating processes underlying the relation between IU and negative emotions. This study is the first to explore the mediating and moderating role of PsyCap and family functioning in the correlation between IU and negative emotions. This study provides a novel perspective on the factors influencing the emotions of students preparing for art college entrance examination during COVID-19. Second, many local and foreign studies on IU focused on a variety of groups and situations. For example, they have investigated IU among pregnant women (75), firefighters (89), and breast cancer patients (28). Few empirical studies have focused on the National College Entrance Examination. This study expands the research scope of IU, reveals an internal mechanism of IU on negative emotions, and contributes to the development of the IU to some extent. Third, this study extended the conservation of resources theory by considering the interaction effect of family functioning and PsyCap on negative emotions. Mental health is associated with PsyCap and family functioning. The interaction effect of PsyCap and family functioning on negative emotions suggests that the process of resource accumulation may lead to a “ceiling effect.” Consequently, the link between PsyCap and negative emotions was weaker in individuals with high family functioning.

Through quantitative analysis, this study obtained important insights on the negative emotions faced by high school art students. Educators must focus on reducing the IU and improving the PsyCap and family functioning of high school art students to reduce the onset of negative emotions among them. Negative emotions can be reduced in three ways: First, IU can be addressed using interventions (90) such as psychoeducation and raising awareness of IU and problem solving in the face of uncertainties. Second, by improving the level of PsyCap, the rise of negative emotions in high school art students can be prevented. The components of PsyCap can be strengthened through interventions (91) such as individual or group psychological counseling, PsyCap lectures, and mental health therapy. Third, the moderating effect of family functioning suggests that by improving family functioning the impact of negative emotions on the students can be reduced. Family functioning lectures and family counseling can enhance the PsyCap and reduce the negative emotions faced by high school art students. The Chinese government promulgated the Law of the People’s Republic of China on the Promotion of Family Education in 2021. However, many parents have not realized the importance of family education; therefore, it is necessary for the government to take some measures to improve the quality of family education. In addition, parents should pay more attention to their children, respect their children’s opinions, and strengthen family cohesion among family members to improve the level of family functioning and alleviate negative emotions.

### 4.5 Limitations and future directions

There are several limitations to this study. First, the cross-sectional approach cannot validate temporal changes or allow causal conclusions across research variables. Future studies can employ longitudinal designs to validate our results because IU and negative emotions may change over time. Second, the self-reported questionnaire survey used in this study may be affected by social desirability, especially for variables with very high social desirability such as negative emotions. Future research should collect data from implicit measures or self-report questionnaires from teachers and classmates. Third, the present study included Chinese high school art students. During the epidemic, the Chinese government adopted stricter epidemic prevention regulations than the governments of other countries. Moreover, high school art students may be more sensitive to emotions than students of other subjects. Future research should collect data from different countries and student groups. Fourth, the present study was conducted during the COVID-19. Future research should be conducted under different conditions. Fifth, this research focused only on the general concept of IU instead of focusing on a specific IU, such as intolerance of COVID-19-related uncertainty or intolerance of examination-related uncertainty. Finally, the mean scores of family functioning and PsyCap were high in our sample, suggesting that most Chinese high school art students have high family functioning and PsyCap. Further studies can examine these relationships in groups with low family functioning and PsyCap.

## 5 Conclusion

In summary, the present study attempted to examine the factors affecting negative emotions among Chinese high school art students during COVID-19. The results suggest a negative association between IU and negative emotions in Chinese high school art students. In addition, PsyCap played a mediating role in this relationship. Moreover, family functioning had a moderating effect on the link between PsyCap and negative emotions. The interaction effect shows that family functioning acts as a protective factor that weakens the negative impact of low PsyCap on emotional well-being. Furthermore, our findings on the association between family functioning and PsyCap provide a knowledge base for future studies on reducing negative emotions during COVID-19.

## Data availability statement

The raw data supporting the conclusions of this article will be made available by the authors, without undue reservation.

## Ethics statement

The studies involving humans were approved by the Ethics Committee of the School of Psychology of Jiangxi Normal University. The studies were conducted in accordance with the local legislation and institutional requirements. Written informed consent for participation in this study was provided by the participants’ legal guardians/next of kin.

## Author contributions

CF: Conceptualization, Data curation, Formal analysis, Methodology, Software, Visualization, Writing – original draft. JL: Conceptualization, Investigation, Resources, Writing – review & editing. BY: Conceptualization, Methodology, Project administration, Writing – review & editing. QY: Investigation, Project administration, Writing – review & editing.
